# Social inequality in health behaviours in cohabitating individuals with CVD: Jes Bak Sørensen

**DOI:** 10.1093/eurpub/ckac131.220

**Published:** 2022-10-25

**Authors:** JB Sørensen, K Friis, NK Nissen, T Maribo, BK Nielsen, CT Mejdahl, AML Thomsen

**Affiliations:** 1 Public Health and Quality Improvement, DEFACTUM, Central Denmark Region, Aarhus N, Denmark; 2 Department of Public Health, Aarhus University, Aarhus, Denmark

## Abstract

**Background:**

Despite a decrease in mortality rates CVD remains the leading cause of morbidity and mortality in Europe. Health behavioural risk factors, low socioeconomic status and cohabitation status are all associated with CVD. However, little is known about social inequality in health behaviour among cohabitating individuals with CVD. Thus, the aim of this study was to examine social inequality in health behaviour among cohabitating individuals with CVD.

**Methods:**

Register data on CVD were linked with self-reported health behaviour from the Danish 2017 population-based health survey ‘How are you?'. In total, 2,443 survey participants aged 45 years and above were registered with CVD. Daily smoking was assessed using a single question about smoking habits. Physical inactivity was categorised as less than 30 minutes of physical activity at least six days per week. Respondents with a BMI ≥ 30 were considered obese. Unhealthy diet was assessed using the Diet Quality Score. Moderate risk alcohol consumption was categorised as exceeding the Danish Health Authority’s recommendations. Self-reported educational attainment was used as a marker of social position and was categorised as low (0-10 years), medium (11-15 years) or high (≥ 15 years). Sociodemographic differences in health behaviour were compared using adjusted logistic regression models with health behaviours as dependent variables and adjusted for sex, age, ethnic background, time since initial CVD diagnosis and multimorbidity.

**Results:**

Cohabitating individuals with CVD and low educational attainment had higher adjusted odds for daily smoking (3.31), physical inactivity (2.10), unhealthy diet (6.37) and obesity (2.55) than cohabitating individuals with CVD and high educational attainment. However, they also had lower adjusted odds for moderate risk alcohol intake (0.35).

**Conclusions:**

Social inequality in daily smoking, physical inactivity, unhealthy diet and obesity was found among cohabitating individuals with CVD.

**Key messages:**

• Social inequality in health behaviour was found among cohabitating individuals with CVD. Thus, low educational attainment affects CVD risk profile regardless of cohabitation status.

• Social inequality in health behaviour among cohabitating individuals with CVD should be addressed in public health strategies, targeted secondary prevention, treatment and rehabilitation.

